# P-635. Clinical Impact and Comparative Outcomes of hMPV and RSV Infections in Hematological Malignancy Patients: A Multinational Cohort Study

**DOI:** 10.1093/ofid/ofaf695.848

**Published:** 2026-01-11

**Authors:** Jon Salmanton-Garcia, Francesco Marchesi, Oliver A Cornely, Livio Pagano

**Affiliations:** University Hospital Cologne, Cologne, Germany, Cologne, Nordrhein-Westfalen, Germany; IRCCS Regina Elena National Cancer Institute, Rome, Lazio, Italy; University of Cologne, Faculty of Medicine and University Hospital Cologne, Cologne, Nordrhein-Westfalen, Germany; Fondazione Policlinico Universitario Agostino Gemelli - IRCCS, Rome, Lazio, Italy

## Abstract

**Background:**

Respiratory viruses cause major morbidity and mortality in hematologic malignancies. While influenza and SARS-CoV-2 are well-characterized, data on hMPV and RSV remain scarce. We compared their clinical features in this high-risk group.

**Methods:**

We conducted a multinational study of 130 hMPV and 243 RSV cases (2023–2024), with matched influenza and SARS-CoV-2 cohorts. Data covered patient characteristics, treatment, costs, and 30-day mortality.

**Results:**

The hMPV and RSV groups had similar demographics and disease profiles, mainly lymphoma and plasma cell disorders. Most infections occurred in winter, with >60% presenting with non-pulmonary symptoms. Severe disease occurred in 52.3% (hMPV) and 63.8% (RSV). Hospitalization rates were high (63.8% vs. 74.9%), ICU admissions similar (18.5% vs. 8.6%), and 30-day mortality was 10.0% (hMPV) vs. 12.8% (RSV).

Compared to hMPV, influenza cases had higher costs (US $33,250 vs. $18,517; p=0.034), greater antiviral use (74.1% vs. 1.9%; p< 0.001), and fewer bacterial co-infections (9.3% vs. 27.8%; p=0.024). SARS-CoV-2 cases also had more antivirals (65.1% vs. 1.2%; p< 0.001), fewer bacterial co-infections (9.3% vs. 25.6%; p=0.008), but slightly less severe disease (44.2% vs. 47.7%; p=0.003).

RSV was linked to more secondary infections than influenza (bacterial 21.3% vs. 12.4%, p=0.033; viral 5.6% vs. 1.1%, p=0.035), higher ward costs, and shorter intermediate care stays (5 vs. 12 days; p=0.010). Influenza patients were more often vaccinated (11.2% vs. 0.6%; p< 0.001) and treated with antivirals (82.0% vs. 15.2%; p< 0.001).

RSV showed more pulmonary symptoms (33.0% vs. 19.2%; p< 0.001), severe cases (62.1% vs. 40.4%; p< 0.001), and hospitalizations (74.4% vs. 54.2%; p< 0.001) than SARS-CoV-2, which had higher intermediate care costs and more targeted therapy (68.5% vs. 14.3%; p< 0.001).

hMPV and RSV had comparable clinical profiles and outcomes, though hMPV showed slightly less treatment and more bacterial co-infections.

**Conclusion:**

In hematologic malignancy patients, hMPV and RSV cause substantial morbidity, high hospitalization and ICU rates, and 30-day mortality (10.0% and 12.8%), comparable to influenza and SARS-CoV-2. Yet, they remain under-treated, highlighting the need for improved diagnostics and targeted care.

**Disclosures:**

Jon Salmanton-Garcia, MSc, MPH, PhD, menarini, gilead, astrazeneca, pfizer: Honoraria Oliver A. Cornely, Prof. Dr., Al-Jazeera Pharmaceuticals/Hikma: Honoraria|Basilea: Advisor/Consultant|Cidara: Advisor/Consultant|Cidara: Board Member|Cidara: Grant/Research Support|Elion: Advisor/Consultant|F2G: Grant/Research Support|Gilead: Advisor/Consultant|Gilead: Grant/Research Support|Gilead: Honoraria|GlaxoSmithKline: Advisor/Consultant|GlaxoSmithKline: Honoraria|Grupo Biotoscana/United Medical/Knight: Honoraria|Melinta: Advisor/Consultant|Melinta: Board Member|MSD: Honoraria|Mundipharma: Advisor/Consultant|Mundipharma: Grant/Research Support|Mundipharma: Honoraria|Pfizer: Advisor/Consultant|Pfizer: Grant/Research Support|Pfizer: Honoraria|Pulmocide: Board Member|Scynexis: Advisor/Consultant|Scynexis: Grant/Research Support|Shionogi: Advisor/Consultant|Shionogi: Honoraria

